# Differential Diagnosis of Solitary Fibrous Tumor/Hemangiopericytoma and Angiomatous Meningioma Using Three-Dimensional Magnetic Resonance Imaging Texture Feature Model

**DOI:** 10.1155/2020/5042356

**Published:** 2020-12-01

**Authors:** Junyi Dong, Meimei Yu, Yanwei Miao, Huicong Shen, Yi Sui, Yangyingqiu Liu, Liang Han, Xiaoxin Li, Meiying Lin, Yan Guo, Lizhi Xie

**Affiliations:** ^1^Department of Radiology, The First Affiliated Hospital of Dalian Medical University, Dalian 116000, China; ^2^Department of Radiology, Beijing Tian Tan Hospital, Capital Medical University, Beijing 100050, China; ^3^Department of Hepatobiliary Surgery, The First Affiliated Hospital of Dalian Medical University, Dalian 116000, China; ^4^Life Science, GE Healthcare, Shenyang 110000, China; ^5^GE Healthcare, MR Research China, Beijing, China

## Abstract

**Background:**

Intracranial solitary fibrous tumor(SFT)/hemangiopericytoma (HPC) is an aggressive malignant tumor originating from the intracranial vasculature. Angiomatous meningioma (AM) is a benign tumor with a good prognosis. The imaging manifestations of the two are very similar. Thus, novel noninvasive diagnostic method is urgently needed in clinical practice. Texture analysis and model building through machine learning may have good prospects.

**Aim:**

To evaluate whether a 3D-MRI texture feature model could be used to differentiate malignant intracranial SFT/HPC from AM.

**Method:**

A total of 97 patients with SFT/HPC and 95 with AM were included in this study. Patients from each group were randomly divided into the train (70%) and test (30%) sets. ROIs were drawn along the edge of the tumor on each section of T1WI, T2WI, and contrasted T1WI using ITK-SNAP software. The segmented image was imported into the AK software for texture feature extraction, and the 3D ROI signal intensity histograms of T1WI, T2WI, and contrasted T1WI were automatically obtained along with all the parameters. Modeling was performed using the language R. Confusion matrix was used to analyze the accuracy of the model. ROC curve was constructed to assess the grading ability of the logistic regression model.

**Results:**

After Lasso dimension reduction, 5, 9, and 7 texture features were extracted from T1WI, T2WI, and contrasted T1WI, respectively; additional 8 texture features were extracted from the combined sequence for modeling. The ROC analyses on four models resulted in an area under the curve (AUC) of 0.885 (sensitivity 76.1%, specificity 87.9%) for T1WI model, 0.918 (73.1%, 95.5%) for T2WI model, 0.815 (55.2%, 93.9%) for contrasted T1WI model, and 0.959 (92.5%, 84.8%) for the combined sequence model and were enough to correctly distinguish the two groups in 71.2%, 81.4%, 69.5%, and 83.1% of cases in test set, respectively.

**Conclusions:**

The radiological model based on texture features could be used to differentiate SFT/HPC from AM.

## 1. Introduction

Intracranial solitary fibrous tumor (SFT)/hemangiopericytoma (HPC) is a rare malignant tumor originating from the intracranial vasculature, which comprises only 1% of all primary central nervous system (CNS) tumors [1]. In the past, it was believed that intracranial SFT/HPC originates from the meninges and thus was considered as a subtype of meningioma [1]. However, with the development of molecular genetics, it was discovered that SFT/HPC originates from arachnoid cap cells [2]. SFT/HPC is an aggressive type of neoplasma, which can easily relapse and metastasize to extracranial tissues.

Angiomatous meningioma (AM) is a rare World Health Organization (WHO) grade I histological subtype of meningioma with a good prognosis, accounting for 2.1% of all meningiomas [3]. AM can be effectively cured through resection. In radiological images, SFT/HPC mimics AM that is usually benign [3]. Therefore, preoperative identification of both is essential.

Screening MRI is the primary method to identify SFT/HPC and AM; yet, considering that images of both tumors are very similar, tumor differentiation can be very challenging. Imaging omics is aimed at maximizing the potential of medical imaging in disease diagnosis through high-dimensional image texture features containing pathophysiological information [4]. Previous studies have shown that texture analysis software can be used to segment the tumor area on the image and perform texture analysis [4–6]; briefly, the characteristic parameters in the image can be extracted for differential comparison, and the tumor imaging heterogeneity can be quantitatively analyzed to provide unrecognizable images by the naked eye. Objective information does not depend on the experience and subjective judgment of the imaging physician and has excellent clinical application value. So far, texture analysis has been applied to identify intracranial tumors [5, 6], grade meningioma [7], and for assessment and survival analysis of the therapeutic response of glioma to chemotherapy [6, 8–10]. More importantly, Kanazawa et al. [11] have suggested that magnetic resonance imaging texture analysis can be useful for distinguishing SFT/HPC from meningioma, especially AM. Still, his study has certain limitations: (1) it was a relatively small sample size retrospective study; (2) this study analyzed only three texture parameters.

This study adopted three-dimensional texture (3D texture) characteristics based on the overall tumor, which can more comprehensively and objectively reflect the heterogeneity of the tumor. The purpose of this study was to further improve the diagnostic levels of these two diseases by using the texture parameters of conventional MRI sequences and to build the models through machine learning.

## 2. Materials and Methods

### 2.1. Patient Selection

The institutional review board approved the current study. The preoperative MRI was performed on 95 patients with AM (47 males and 48 females; mean age: 51.54 ± 11.54 years) and 97 with SFT/HPC (47 males and 50 females; mean age: 42.97 ± 14.35 years) at our institution from May 2012 to March 2019. All MRI results were retrospectively analyzed.

### 2.2. Data Acquisition

All MR images were obtained with a 3.0 T MR imager (Signa HDxt; GE Medical Systems, Milwaukee, WI) with an eight-channel head coil. The imaging protocol included unenhanced axial and sagittal T1-weighted sequences, axial and coronal T2-weighted sequences, and contrast-enhanced axial, sagittal, and coronal T1-weighted sequences. The scanning parameters were T1WI (TR/TE, 350 msec/9 msec); T2WI (TR/TE, 3,500 msec/110 msec); thickness, 6.0 mm; spacing, 1.0 mm; FOV, 220 × 220 mm; matrix, 448 × 256; sagittal and coronal slice, 8.0 mm; and layer spacing, 2.0 mm. An enhanced scan bolus Gd-DTPA (DTPA magnetic display) was given intravenously at a concentration of 0.1 mmol/kg body weight with a flow rate of 3 ml/sec.

### 2.3. Image Processing

**Figure 1:**
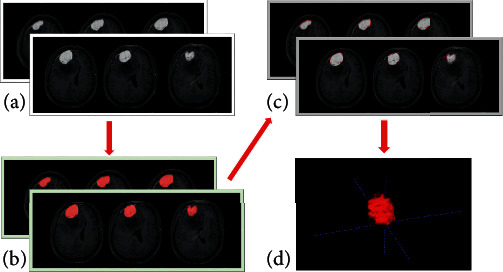
(a) Contrasted T1WI. (b) Contrasted T1WI image generated by ITK-SNAP software to depict the ROI of the tumor. (c) 3D ROI image of the tumor (red area) that is calculated to superimpose at all levels in the contrasted T1WI map. (d) A three-dimensional image of the tumor.

**Figure 2:**
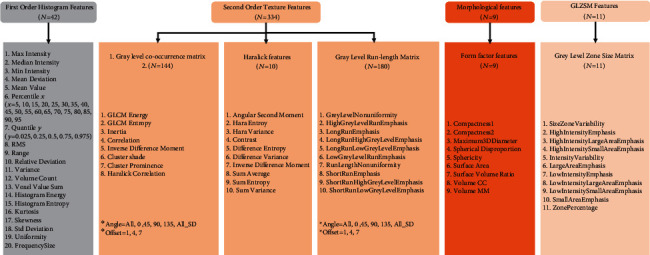
A total of 396 texture parameters extracted by AK software.

First, based on image segmentation of the whole tumor, all T1WI, T2WI, and contrasted T1WI data with Digital Imaging and Communication in Medicine (DICOM) format were transferred from the picture archiving and communication system (PACS) workstation (Centricity PACS 3.1.1.4, GE Healthcare) to ITK-SNAP software. Two radiologists (residents and deputy chief physicians), who were blind to the grouping, manually selected the regions of interest (ROIs) along the edge of the tumor parenchyma on the contrasted T1WI, T1WI, and T2WI images; T2WI and contrast-enhanced T1WI were used as a reference to determine tumor areas. The ROIs were then manually drawn along the margin of the tumor parenchyma in each slice, with the intent to encompass the whole tumor volume. Consequently, the ROIs of all layers were merged into a 3D ROI (see Figure 1). Finally, the segmented image was imported into the AK (Artificial intelligence kit) software for texture feature extraction, and the 3D ROI signal intensity histograms of T1WI, T2WI, and contrasted T1WI were automatically obtained along with all the parameters (see Figure 2).

### 2.4. Statistical Methods and Modeling

Modeling was performed using the language R (RStudio Version 1.0.143–© 2009-2016 RStudio, Inc.). Approx. 70% of cases from each group were classified into the train set (133 cases); AM group (66 cases) and SFT/HPC group (67 cases) were used to establish the model. The remaining 30% were classified into the test set (59 cases), AM group (29 cases) and SFT/HPC group (30 cases), to verify the accuracy of the established model.

A comparison of texture features in T1WI sequences was analyzed using independent sample *t*-test and Kruskal-Wallis test; a *P* value < 0.05 was considered statistically significant. Univariate logistic regression analysis (*P* < 0.05) and Spearman's correlation analysis (*P* ≥ 0.05 or *P* < 0.05, *r* < 0.9) were used to screen for the parameters with high predictive power. T2WI and contrasted T1WI sequence texture feature used the Lasso method to reduce dimensionality and selected high-performance parameters. Parameters with high predictive power in the three sequences were further eliminated using the stepwise iterative method, and the remaining high-performance parameters were fed into a multivariate logistic regression analysis to determine an optimal logistic regression model for tumor classification. The confusion matrix was used to analyze the accuracy of the model. ROC curve was constructed to assess the grading ability of the logistic regression model.

## 3. Results

### 3.1. Establishment of T1WI, T2WI, and Contrasted T1WI Texture Feature Models

After applying the dimension reduction and stepwise iterative method, the high-performance parameters of the T1WI texture feature model were kurtosis (KU), skewness (SK), stdDeviation (ST), GLCMEntropy_angle90_offset1 (GLCME_90-1_), and SmallAreaEmphasis (SAE). The T1WI texture feature modeling formula was the following:

(1) $$\begin{array}{c} f\left(\text{T}1\text{WI}\right)=-13.4078+0.3066\times \text{KU}-1.2143\times \text{SK}+0.0351\times \text{ST}-1.1441\times {\text{GLCME}}_{90‐1}+23.9030\times \text{SAE}. \end{array}$$

**Figure 3:**
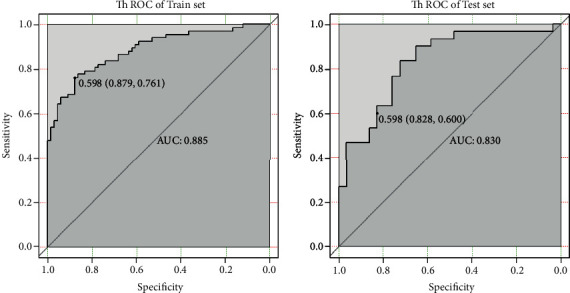
T1WI texture feature model for identification of HPC and AM performance.

In the train set, the ACC of the T1WI model in identifying SFT/HPC and AM was 0.820, and the area under the ROC curve (AUC) was 0.885, with the cutoff value of 0.598, a sensitivity of 76.1%, and a specificity of 87.9%. In the test set, ACC was 0.712 and AUC was 0.830, with a cutoff value of 0.598, a sensitivity of 60.0%, and a specificity of 82.8% (see Figure 3).

The high-performance parameters of the T2WI texture feature model were GLCMEnergy_AllDirection_offset1_SD (GLCME_A-1-SD_), Inertia_angle90_offset7 (IN_90-7_), InverseDifferenceMoment_AllDdirection_offset7_SD (IDM_A-7-SD_), LongRunLowGreyLevelEmphasis_AllDirection_offset4_SD (LRLGLE_A-4-SD_), LowGreyLevelRunEmphasis_AllDirection_offset7_SD (LGLRE_A-7-SD_), ShortRunEmphasis_angle135_offset1 (SRE_135-1_), ShortRunHighGreyLevelEmphasis_angle90_offset4 (SRHGLE_90-4_), HighIntensitySmallAreaEmphasis (HISAE), and LowIntensityLargeAreaEmphasis (LILAE). The T2WI texture feature modeling formula was the following:

(2) $$\begin{array}{c} f\left(\text{T}2\text{WI}\right)=9.78{\text{e}}^{2}+1.23{\text{e}}^{8}\times {\text{GLCME}}_{\text{A}‐1‐\text{SD}}-2.81{\text{e}}^{-3}\times {\text{IN}}_{90‐7}-2.53{\text{e}}^{4}\times {\text{IDM}}_{\text{A}‐7‐\text{SD}}+8.56{\text{e}}^{-1}\times {\text{LRLGLE}}_{\text{A}‐4‐\text{SD}}-9.72{\text{e}}^{10}\times {\text{LGLRE}}_{\text{A}‐7‐\text{SD}}-9.79{\text{e}}^{2}\times {\text{SRE}}_{135‐1}-1.38{\text{e}}^{-4}\times {\text{SRHGLE}}_{90‐4}+9.66{\text{e}}^{-7}\times \text{HISAE}+8.04{\text{e}}^{4}\times \text{LILAE}. \end{array}$$

**Figure 4:**
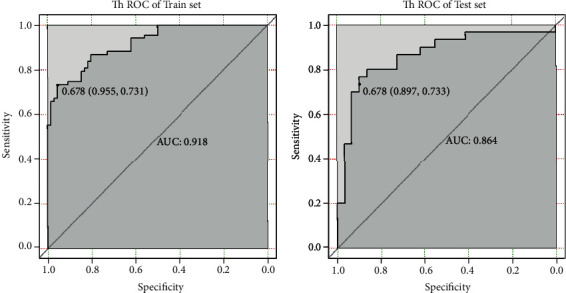
T2WI texture feature model for identification of HPC and AM performance.

In the train set, the ACC of T2WI model in identifying SFT/HPC and AM was 0.842, and the area under the ROC curve (AUC) was 0.918, with a cutoff value of 0.678, a sensitivity of 73.1%, and a specificity of 95.5%. In the test set, ACC was 0.814 and AUC was 0.864, with a cutoff value of 0.678, a sensitivity of 73.3%, and a specificity of 89.7% (see Figure 4).

The high-performance parameters involved in the contrasted T1WI texture feature model were Quantile_0.025_, RelativeDeviation (RD), VoxelValueSum (VVS), ClusterProminence_AllDirection_offset1_SD (CP_A-1-SD_), GLCMEntropy_AllDirection_offset7_SD (GLCME_A-1-SD_), LongRunHighGreyLevelEmphasis_AllDirection_offset1_SD (LRHGLE_A-1-SD_), and ShortRunHighGreyLevelEmphasis_AllDirection_offset4_SD (SRHGLE_A-4-SD_). The contrasted T1WI texture feature model modeling formula was the following:

(3) $$\begin{array}{c} f\left(\text{contrastedT}1\text{WI}\right)=-6.05{\text{e}}^{-1}+2.84{\text{e}}^{-3}\times {\text{Quantile}}_{0.025}+8.38{\text{e}}^{-1}\times \text{RD}+3.12{\text{e}}^{-8}\times \text{VVS}-1.68{\text{e}}^{-14}\times {\text{CP}}_{\text{A}‐1‐\text{SD}}+2.89{\text{e}}^{-1}\times {\text{GLCME}}_{\text{A}‐1‐\text{SD}}-1.05{\text{e}}^{-4}\times {\text{LRHGLE}}_{\text{A}‐1‐\text{SD}}-1.07{\text{e}}^{-5}\times {\text{SRHGLE}}_{\text{A}‐4‐\text{SD}}. \end{array}$$

**Figure 5:**
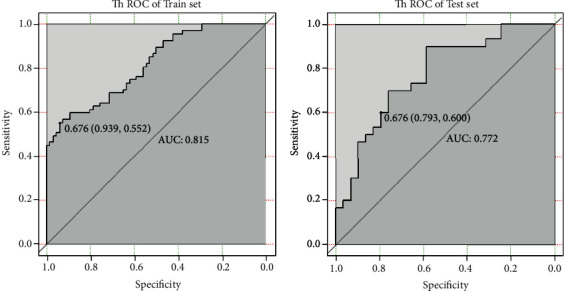
Contrasted T1WI texture feature model for identification of HPC and AM performance.

In the train set, the ACC of contrasted T1WI model in identifying SFT/HPC and AM was 0.744, and the area under the ROC curve (AUC) was 0.815, which had a cutoff value of 0.676, a sensitivity of 55.2%, and a specificity of 93.9%. In the test set, ACC was 0.695 and AUC was 0.772, which had a cutoff value of 0.676, a sensitivity of 60.0%, and a specificity of 79.3% (see Figure 5).

### 3.2. Establishment of Total Sequences Combine Texture Feature Model

After the dimension reduction and stepwise iterative method, the high-performance parameters of the total sequences combined texture feature model were Percentile_95T1WI_, KU_T1WI_, GLCMEntropy_angle90_offset1(GLCME_90-1_)_T1WI_, HaraEntroy(HE)_contrasted T1WI_, RunLengthNonuniformity_angle90_offset7(RLNU_90-1_) _contrasted T1WI_, Range(RA)_T2WI_, ClusterProminence_angle135_offset4(CP_135-4_)_T2WI_, and LongRunHighGreyLevelEmphasis_angle45_offset4(LRHGLE_45-4_)_T2WI_. The total sequences combined model modeling formula was the following:

(4) $$\begin{array}{c} f_{\left(\text{total sequences combine}\right)}=-8.91+1.93{\text{e}}^{-2}\times \text{P}{\text{ercentile}}_{95\left(\text{T}1\text{WI}\right)}+3.09{\text{e}}^{-1}\times {\text{KU}}_{\left(\text{T}1\text{WI}\right)}-1.22\times {\text{GLCME}}_{90-1\left(\text{T}1\text{WI}\right)}+2.56{\text{e}}^{1}\times {\text{HE}}_{\left(\text{contrasted T}1\text{WI}\right)}+3.14{\text{e}}^{-5}\times {\text{RLNU}}_{90-1\left(\text{contrasted T}1\text{WI}\right)}-3.76{\text{e}}^{-3}\times {\text{RA}}_{\left(\text{T}2\text{WI}\right)}+1.01{\text{e}}^{-8}\times {\text{CP}}_{135‐4\left(\text{T}2\text{WI}\right)}-1.41{\text{e}}^{-4}\times {\text{LRHGLE}}_{45‐4\left(\text{T}2\text{WI}\right)}. \end{array}$$

**Figure 6:**
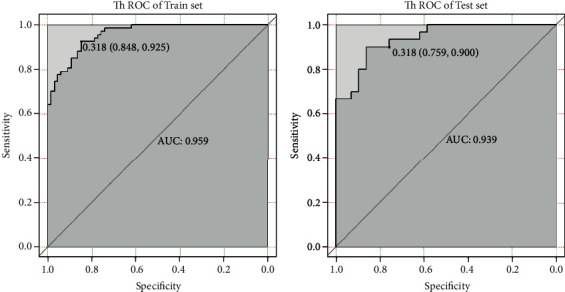
Total sequences combine the texture feature model for identification of HPC and AM performance.

According to ROC analysis, the combined model used to identify AM and SFT/HPC in the train set had an AUC of 0.959 (cutoff value = 0.318, specificity of 84.8%, and sensitivity of 92.5%), and the accuracy of the combined model was 0.887. In the test set, AUC was 0.939, with a cutoff value of 0.318, a sensitivity of 90.0%, and a specificity of 75.9%, and the accuracy of the combined model was 0.831 (see Figure 6).

Finally, the AUC, ACC, cut-off, sensitivity, and specificity of the four models are summarized in Table 1.

## 4. Discussion

Image segmentation is a critical session for the MRI images to be used in brain tumor studies. In recent years, semiautomatic and fully automatic algorithms for brain tumor segmentation have been developed rapidly. A study presented a fully automatic brain tumor detection and segmentation method using the U-Net based deep convolution network and demonstrated that this method can provide both efficient and robust segmentation compared to manual delineated ground truth [12]. Soltaninejad et al. [13] proposed a supervised learning based method for segmentation tumour in multimodal MRI brain images. Supervoxels were calculated using information fusion from multimodal MRI images, which also demonstrated promising results in the segmentation of brain tumor. Even so, there are still several opening challenges for this task mainly due to the high variation of brain tumors in size, shape, regularity, location, and their heterogeneous appearance. In addition, AM and HPC/SFP are rare diseases, and the data is relatively rare compared to common diseases. We have certain reasons to believe that segmentation based on big data may have certain errors. Considering the above reasons, the segmentation was still relied on manual delineation by human operators in this study.

In this study, radiomics method was used to construct four models to identify the 3D-texture features of SFT/HPC and AM based on conventional MRI sequence images, including the T1WI model, T2WI model, contrasted T1WI model, and a combined sequence model. Briefly, the combined sequence model showed the best performance, followed by the T2WI model. As a noninvasive predictive method, all four models can provide reference information for preoperative treatment planning and patient prognosis. Due to the relatively large number of cases, we have established a relatively accurate MRI radiological model for preoperative identification of SFT/HPC and AM. To the best of our knowledge, this is the first study that established an MRI radiological model, which can be used to differentiate SFT/HPC from AM.

Texture features are essential markers for intratumoral homogeneity. Among the twenty-three texture features that were involved in building our models, eight were histogram-based features (KU, SK, ST, RD,VVS, RA, Quantile_0.025_, and Percentile_95_), and twelve were matrix-based features, including five GLCM features (GLCMEnergy, GLCMEntropy, IN, IDM, and CP) and one Haralick feature (HE). Besides, there were six GLRM features (LRHGLE, LRLGLE, LGLRE, RLNU, SRE, and SRHGLE), and three GLZSM features (HISAE, LILAE, SAE). Histogram-based features are first-order statistics that primarily rely on intensity information (or brightness information) within and around the tumor. These features are used to investigate the overall distribution of intensity information within and around the tumor. For example, “kurtosis” is a measure of the “tailedness” of the median distribution of image ROI, which can be used to describe the concentration of image brightness information. Higher kurtosis means that the mass of the distribution is concentrated at the tail. “Skewness” represents the measure of “skewness” of the median distribution of the image ROI and is used to describe the degree of asymmetric distribution in the histogram. The percentile (%) of a distribution is defined as the brightness value. IDM represents the uniformity of pixel signal strength in the image, which can reflect the heterogeneity of tumor tissues.

Matrix-based features are second-order statistics that can be used to analyze the complexity within the tumor and around the tumor, changes in the hierarchy, and thickness of the texture. For example, inertia reflects the clarity of the image and texture groove depth. The contrast is proportional to the texture groove; high groove values produce more clarity, while small values lead to small contrast and fuzzy image. GLCMEntropy measures the average amount of information required to encode an image value. SRHGLE measures the joint distribution of shorter run lengths with higher grey-level values. Larger value leads to a more complex image and smaller image grey value. LRLGLE measures the joint distribution of longer-run lengths with lower grey-level values. SRE is a measure of short lengths, with larger values representing shorter lengths and finer textures. GLZSM is particularly efficient to characterize the texture homogeneity, nonperiodicity, or speckle like texture.

So far, many studies have reported the use of radiological models based on the texture features of CT and MRI images for the identification/differentiation of tumors. Chen et al. [14] found that the radiomics model based on contrast-enhanced computed tomography (CECT) could be used for predicting acute pancreatitis (AP) recurrence. As a quantitative method, radiomics exhibits promising performance in alerting relapsed patients to potential preventive measures. Kang et al. [15] tested the technical feasibility, generalizability, and diagnostic performance of a radiomics model using ADC maps for identification of atypical primary central nervous system lymphoma (PCNSL) mimicking glioblastoma. His model showed good generalizability and improved diagnostic performance than single-parameter measurements in identifying atypical PCNSL mimicking glioblastoma by providing robust high-dimensional analyses of conventional and physiological imaging features. Furthermore, Chen and colleagues [16] confirmed that an MRI-based combined radiography nomogram can effectively predict the immune score of HCC and help to make treatment decisions.

Our study showed that the combined sequence model was superior to any single sequence model in differentiating SFT/HPC from AM. T2WI sequence is the most commonly used sequence to evaluate brain pathology and the degree of tumor invasion. T1WI and contrasted T1WI sequences provide anatomical information, while tissue enhancement reflects increased blood-brain barrier permeability [17]. Considering that each sequence has different functions, combining multiple sequences may improve the accuracy in differentiating SFT/HPC from AM. Also, Tian et al. [18] verified the superiority of radiomics features extracted by multiparameter MRI in glioma grading and found that the combined application of multiparameter MRI has higher classification efficiency, which was consistent with our data. It is worth noting that in the three sequences of conventional MRI, the AUC of the radiological model based on T2WI image texture features was higher than in the other two sequences. One explanation for this may be that the T2WI sequence has a relatively long echo time and high contrast between tissues, so the image contains many differential texture features with discriminative value. Among the related studies on breast, one study suggested that T2WI images have a significant role in the differentiation of benign and malignant diseases of nonmass breast tumors [19]. Li et al. [20] confirmed that texture features of SPAIR T2W-MRI can be classified into three different types of single-liver lesions and may serve as an adjunct tool for accurate diagnosis of these diseases. Surprisingly, we also found that the contrasted T1WI model had the lowest AUC among the three conventional MRI sequence models. Furthermore, Zhang et al. [21] found that T1w+Gd had the lowest AUC in all MRI sequences when evaluating the feasibility of texture analysis on preoperative conventional MRI images in predicting early malignant transformation from low- to high-grade glioma, which was consistent with our results. Nevertheless, T1WI+C were very useful for visual evaluation of tumors.

## 5. Conclusions

The radiological model based on texture features could be used to differentiate SFT/HPC from AM. Besides, our texture analysis results, which extract many quantitative features from various kinds of digital images, provide the basis for further radiomics analyses and are a rapidly expanding research area [22, 23].

### 5.1. Limitations of the Study

Limitations of this study must be addressed. (1) This study was a retrospective study, which means that further prospective studies of a larger range of patients and multivariate analysis are necessary to verify these results. (2) In this study, only the parenchymal part of the tumor was selected for texture analysis. The peritumoral edema area of the two tumors was not analyzed, and the MRI signs were further combined with the texture parameters to improve the discrimination efficiency. (3) The ROIs were manually determined. Automatic segmentation algorithms may facilitate the procedure. (4) The correlation between the significance of various parameters of texture analysis and the biological mechanism of tumors was still insufficient; thus, further research is required to confirm our findings.

## Figures and Tables

**Table 1:** AUC, ACC, cut-off, sensitivity, and specificity of the four texture feature model.

Task	ACC	AUC	Cut-off	Sensitivity	Specificity
Train (T1WI)	0.820	0.885	0.598	76.1%	87.9%
Test (T1WI)	0.712	0.830	0.598	60.0%	82.8%
Train (T2WI)	0.842	0.918	0.678	73.1%	95.5%
Test (T2WI)	0.814	0.864	0.678	73.3%	89.7%
Train (contrasted-T1WI)	0.744	0.815	0.676	55.2%	93.9%
Test (contrasted-T1WI)	0.695	0.772	0.676	60.0%	79.3%
Train (combined)	0.887	0.959	0.318	92.5%	84.8%
Test (combined)	0.831	0.939	0.318	90.0%	75.9%

## Data Availability

The data of this study are available from the corresponding author upon request.
